# The quality of antimicrobial prescribing in acute care hospitals: results derived from a national point prevalence survey, Germany, 2016

**DOI:** 10.2807/1560-7917.ES.2019.24.46.1900281

**Published:** 2019-11-14

**Authors:** Seven Johannes Sam Aghdassi, Frank Schwab, Sonja Hansen, Luis Alberto Peña Diaz, Michael Behnke, Petra Gastmeier, Tobias Siegfried Kramer

**Affiliations:** 1 Charité-Universitätsmedizin Berlin, corporate member of Freie Universität Berlin, Humboldt-Universität zu Berlin and Berlin Institute of Health, Institute of Hygiene and Environmental Medicine, Berlin, Germany; 2 National Reference Centre for Surveillance of Nosocomial Infections, Berlin, Germany

**Keywords:** antimicrobial stewardship, antimicrobial use, epidemiology, surveillance, point prevalence survey

## Abstract

**Background:**

Robust data on the quality of antimicrobial prescriptions in German acute care hospitals are scarce. To establish and implement antimicrobial stewardship (AMS) measures and to increase prudent antimicrobial use (AMU), the identification of appropriate process and quality indicators is pertinent.

**Aim:**

Our main objective was to identify parameters associated with adequate AMU and inadequate AMU by analysing point prevalence data. Our secondary goal was to describe the current state of AMS implementation in Germany.

**Methods:**

A national point prevalence survey for healthcare-associated infections and AMU was conducted in German hospitals in 2016. Data on structure and process parameters were also collected. Recorded antimicrobial prescriptions were divided into adequate, inadequate and undefinable AMU. A multivariable linear regression analysis was performed to examine the correlation of selected structure and process parameters with the adequacy of recorded antimicrobials.

**Results:**

Data from 218 acute care hospitals, 64,412 patients and 22,086 administered antimicrobials were included. Multivariable linear regression analysis revealed that documentation of a reason for AMU in the patient notes increased the likelihood of adequate AMU and decreased the likelihood of inadequate AMU significantly (p < 0.001), while tertiary care hospital type had the opposite effect (p < 0.001).

**Conclusion:**

Through associating structural and process parameters with adequacy of AMU, we identified parameters that increased the odds of prudent AMU. Documentation was a key element for improving AMU. Revealed deficits regarding the implementation of AMS in German hospitals concerning dedicated staff for AMS activities and establishment of regular AMU training and AMU audits should be tackled.

## Introduction

Healthcare systems worldwide have attempted to establish antimicrobial stewardship (AMS) programmes since the concept of AMS was introduced over 20 years ago [1]. AMS focuses on improving prescription practices of antimicrobials to improve outcomes in patients with infectious diseases through more effective treatment, and to reduce adverse effects. Such aims have been shown to be achievable for instance, through reduction and timely discontinuation of ineffective or prolonged antimicrobial treatments [2]. Recent data on the prevalence of antimicrobial use (AMU) in German hospitals revealed a shift in the most frequently administered antimicrobials to a higher proportion of broad-spectrum antimicrobials [3], which generally are associated with more adverse effects than antimicrobials with a narrower spectrum.

To promote prudent use of antimicrobials in German hospitals, various measures have been proposed [4-6]. In 2013, a national guideline on the prudent use of antimicrobials in hospitals was published [7]. Among other measures, the guideline recommended the implementation of interdisciplinary AMS teams with designated staff in every hospital. Many hospitals in Germany have introduced some features of AMS, but the level of implementation varies widely [8].

The term ‘never events’ is used to describe errors that should never occur in medical practice [9]. Although identifying never events for antimicrobials poses a challenge, attempts have been made in this respect [10,11]. Defining inadequate AMU (i.e. never events) from point prevalence data can be a means to achieve this goal.

The main objectives of this study were to describe and analyse the current state of antimicrobial prescription quality in German acute care hospitals that participated in the national point prevalence survey (PPS) of healthcare-associated infections (HAI) and AMU in 2016, and to associate the data with a set of structural and process parameters, which were recorded as part of the survey. Furthermore, as a secondary objective, we aimed to describe the current state of AMS implementation in German acute care hospitals.

## Methods

A cross-sectional PPS was conducted in acute care hospitals in Germany between May and June 2016. All 1,462 hospitals participating in the German nosocomial infection surveillance system ‘Krankenhaus-Infektions-Surveillance-System’ (KISS) as of the first quarter of 2016, and other acute care hospitals in Germany were invited to participate on a voluntary basis.

### Data collection

Data collectors were local hospital staff trained in methodology and HAI definitions at special one-day courses, to ensure methodological consistency. The data were collected according to the methodology and definitions provided by the European Centre for Disease Prevention and Control (ECDC). The light protocol version 5.1 of the ECDC PPS protocol was implemented [12].

All data gathered in the PPS and used in our analyses were from variables included in the ECDC PPS protocol, such as data on HAI and AMU, patient-related data for patients with an active HAI and/or receiving at least one antimicrobial on the day of the survey, as well as structural and process parameters at the hospital and ward level. The original data collection sheets used and information on all data collected can be found in the ECDC PPS protocol [12]. Additional variables, which were not outlined in the protocol, were not collected. Specifics of data collection and management of German PPS data were described previously in more detail [13].

For AMU, the Anatomical Therapeutic Chemical Classification System of the World Health Organization [14] was used. Data on antimicrobials’ route of application, indication for use, duration of application, documentation of a reason for AMU in the patient notes, modification of treatment and dosage, were recorded.

### Analyses of antimicrobial use

While the primary endpoint of the PPS was to estimate the prevalence of patients with HAI, the prevalence of patients with AMU was a secondary endpoint. Collection of data concerning AMU and structural characteristics of participating hospitals allowed further AMU-related analyses. In our study, we investigated the quality of antimicrobial prescriptions and possible associations with hospitals’ structural and process parameters, as specified by the ECDC protocol [12]. In order to do this, the adequacy of every antimicrobial application was determined and allocated to one of three categories: adequate, inadequate or undefinable. This allocation was based on available literature, as specified in Box 1.

Box 1 **Definitions of antimicrobial applications’ adequacy^a^**

Antimicrobial applications defined as **adequate**:

• surgical prophylaxis for not more than 24 hours [6,33],

• AMU for treatment of community-acquired pneumonia with a treatment duration of 7 days or shorter [6,34], and

• AMU for treatment of pyelonephritis with de-escalation of the initial treatment or switch to oral treatment [35,36].

Antimicrobial applications defined as **inadequate**:

• surgical prophylaxis for more than 24 hours [6,33],

• AMU for treatment of community-acquired pneumonia with a treatment duration of more than 7 days [6,34,37],

• AMU for treatment of pyelonephritis without de-escalation of the initial treatment or switch to oral treatment [35,36],

• AMU for treatment of asymptomatic bacteriuria [6,7,36], and

• AMU without clear (i.e. unknown) indication [6,7].

All other antimicrobial applications were considered **undefinable**.

AMU: antimicrobial use.
^a^ Based on available literature.

We then calculated the rate of adequate AMU as the number of adequate antimicrobial applications per 100 definable (i.e. adequate plus inadequate) applications, thereby excluding undefinable AMU from the denominator. In an analogous manner, we calculated the rate of inadequate AMU as the number of inadequate antimicrobial applications per 100 definable applications.

Further analyses were performed to identify structural and process parameters associated with an increase in the rate of adequate or inadequate AMU. To determine either outcome, we performed a univariable and a multivariable linear regression analysis. The multivariable analysis was conducted by variable selection stepwise forward, with a p value < 0.05 for a parameter to be included in the model and a p value < 0.06 for a parameter to remain in the model.

The structural and process parameters included in the univariable analysis are given in Box 2. Parameters were also included in the multivariable linear regression analysis, except those where datasets were incomplete. The parameters hospital type and hospital ownership were analysed as dummy-coded parameters in the univariable and multivariable analyses.

Box 2 **Parameters included in the univariable and multivariable linear regression analyses^a^**

The following structural and process parameters were included in the analyses:

• hospital size (i.e. number of beds: < 300, ≥ 300),

• bed occupancy (%) on the day of the PPS and as a yearly mean (i.e. number of patient days per year divided by number of available hospital bed-days per year),

• hospital type^b^ (primary care, secondary care, tertiary care, specialised hospital),

• hospital ownership^b^ (public, private (not for profit), private (for profit), other/unknown),

• number of blood cultures per 100 patient days,

• number of stool samples for CDI per 100 patient days,

• participation in surveillance networks for CDI,

• participation in surveillance networks for antimicrobial consumption and resistance,

• implementation of key bundles and multimodal strategies for selected AMS aspects,

• percentage of beds with systematic review routines for prescribed antimicrobials within 72 hours,

• equipment with designated AMS personnel,

• prevalence of patients with AMU, and

• percentage of administered antimicrobials with a reason documented in the patient notes.

AMS: antimicrobial stewardship; AMU: antimicrobial use; CDI: *Clostridioides difficile* infection; ECDC: European Centre for Disease Prevention and Control; PPS: point prevalence survey.
^a^ The following parameters where datasets were incomplete were not used in the multivariable linear regression analysis: number of blood cultures per 100 patient days, number of stool samples for CDI per 100 patient days, percentage of beds with systematic review routines for prescribed antimicrobials within 72 hours and equipment with designated AMS personnel.
^b^ The parameters hospital type and hospital ownership were collected in alignment with the ECDC PPS protocol [12] and analysed as dummy-coded parameters in the univariable and multivariable analyses.

### Ethical approval

The German Protection against Infection Act (Infektionsschutzgesetz) requires hospitals in Germany to collect surveillance data on HAI and AMU. All data used in our analyses and presented in this study are surveillance-based data. Ethical approval or informed consent were therefore not required.

## Results

A total of 218 hospitals participated in the PPS (Table 1). Data from 64,412 patients and 22,086 prescribed antimicrobials were included in the analyses. The overall prevalence of patients receiving at least one antimicrobial was 26%. Only 61 hospitals (28%) stated that there was designated AMS staff employed at the hospital site, with nine hospitals reporting one or more AMS full-time equivalent staff members (FTE). A mean number of 0.1 AMS FTE per 250 hospital beds were recorded (median 0.0). A descriptive analysis of structural and process parameters of AMU can be found in Table 2.

**Table 1:** Structural characteristics of hospitals participating in the point prevalence survey, Germany, 2016 (n = 218)

Variable	Group/parameter	Number or median	% or IQR
Hospital type	Primary care	118	54.1
	Secondary care	41	18.8
	Tertiary care	36	16.5
	Specialised hospital	23	10.6
Hospital ownership	Public	103	47.2
	Private, not for profit	63	28.9
	Private, for profit	31	14.2
	Other/unknown	21	9.6
Region in Germany	East^a^	43	19.7
	South-east^b^	27	12.4
	South-west^c^	56	25.7
	North^d^	29	13.3
	West^e^	63	28.9
Hospital size	< 300 beds	105	48.2
	≥ 300 beds	113	51.8
Patient days	Patient days per year	81,586	46,448.0–144,014.0
Bed occupancy per 100 beds	On the day of survey	75	68.5–82.0

IQR: interquartile range.

^a^ Berlin, Brandenburg, Saxony, Saxony-Anhalt and Thuringia.

^b^ Bavaria and Hesse.

^c^ Baden-Württemberg, Saarland and Rhineland-Palatinate.

^d^ Bremen, Hamburg, Lower Saxony, Mecklenburg-West Pomerania and Schleswig-Holstein.

^e^ North Rhine-Westphalia.

**Table 2:** Structural and process parameters of antimicrobial use and antimicrobial stewardship at hospitals participating in the point prevalence survey, Germany, 2016 (n = 218)

Variable	Group/parameter	Number or median	% or IQR
Microbiological diagnostics	Number of blood cultures per 100 patient days	2.1	1.3–3.1
	Number of stool samples for *Clostridioides difficile* infection per 100 patient days	0.7	0.5–1.1
Surveillance (regional or national network)	*C. difficile* infection	119	54.6
	Antimicrobial consumption	83	38.1
	AMR	56	25.7
Components of multimodal strategies (at the hospital level)	Guideline for AMU	157	72.0
	Training for AMU	37	17.0
	Bundle for AMU	136	62.4
	Checklist for AMU	25	11.5
	Audit for AMU	74	33.9
	Surveillance of AMU	145	66.5
	Feedback of data on AMU to frontline HCW	150	64.2
Post-prescription review of antimicrobials within 72 hours	Percentage of hospital beds	0	0.0–16.8
Designated staff for antimicrobial stewardship	Hospital with designated staff for AMS	61	28.0
	Full-time equivalents per hospital	0	0.0–0.1
	Full-time equivalents per 250 beds	0	0.0–0.1

AMR: antimicrobial resistance; AMS: antimicrobial stewardship; AMU: antimicrobial use; HCW: healthcare workers; IQR: interquartile range.

Following the definitions in Box 1, 3,349 (15 %) adequate and 3,872 (18 %) inadequate antimicrobial applications were recorded. Additionally, 14,865 (67 %) applications remained undefinable (i.e. not attributable to the group of adequate or inadequate applications) (Table 3).

**Table 3:** Antimicrobial use in hospitals participating in the point prevalence survey, Germany, 2016 (n = 218)

Variable	Group/parameter	Number or median	% or IQR
Number of recorded antimicrobials	All hospitals	22,086	100
	Per hospital	64.5	38.0–125.0
Number of observed patients	All hospitals	64,412	100
	Per hospital	220.5	122.0–377.0
Prevalence of patients with AMU	All hospitals	26.2	19.5–30.5
Documentation of a reason for AMU in the patient notes	All hospitals	15,165	68.7
	Per hospital	41.5	20.0–85.0
	Per 100 recorded antimicrobials	77.5	54.3–90.9
Adequate applications of antimicrobials	All hospitals	3,349	15.2
	Per hospital	12	4.0–21.0
	Per 100 antimicrobials	16	9.0–24.6
	Per 100 definable antimicrobials	55.3	35.8–71.4
Inadequate applications of antimicrobials	All hospitals	3,872	17.5
	Per hospital	9	4.0–21.0
	Per 100 antimicrobials	16.7	10.5–23.5
	Per 100 definable antimicrobials	50	35.4–66.7
Undefinable applications of antimicrobials	All hospitals	14,865	67.3
	Per hospital	41	24.0–83.0
	Per 100 antimicrobials	67.1	59.3–74.8

AMU: antimicrobial use; IQR: interquartile range.

Results of the multivariable linear regression analysis for an increase in the rate of adequate AMU and inadequate AMU are illustrated in Table 4; corresponding univariable analyses can be found in the supplementary material (Table S1, Table S2). Documentation of a reason for AMU in the patient notes was a parameter associated with both a significant increase in the rate of adequate AMU and a significant decrease in the rate of inadequate AMU. Tertiary care hospital type showed the opposite association.

**Table 4:** Multivariable linear regression analysis by rates of adequate and inadequate antimicrobial applications per 100 definable antimicrobial applications of hospitals participating in the point prevalence survey, Germany, 2016 (n = 218)

Outcome	Parameter	p value	Regression coefficient (95% CI)
Rate of adequate antimicrobial applications per 100 definable applications	Documentation of a reason for AMU in the patient notes (per increase of 1%)	< 0.001	0.22 (0.10 to 0.34)
	Bed occupancy as a yearly mean (per increase of 1%)	0.046	-0.32 (-0.63 to -0.01)
	Tertiary care hospital type	0.001	-14.51 (-22.78 to -6.24)
Rate of inadequate antimicrobial applications per 100 definable applications	Checklist for antimicrobial use (at the hospital level)	0.018	-11.54 (-21.09 to -2.00)
	Documentation of a reason for AMU in the patient notes (per increase of 1%)	< 0.001	-0.23 (-0.34 to -0.11)
	Tertiary care hospital type	< 0.001	14.80 (6.57 to 23.03)

AMU: antimicrobial use; CI: confidence interval.

## Discussion

Key components of AMS are the identification and reduction of improper antimicrobial prescriptions [2,15,16]. In our study, we were able to distinguish adequate from inadequate antimicrobial prescriptions using point prevalence data and to associate adequate and inadequate AMU with structural and process parameters.

Through our multivariable linear regression analysis, documentation of a reason for AMU in the patient notes was identified as a factor to both increase the likelihood of adequate AMU and decrease the risk of inadequate AMU. We consider this to be our most conclusive and relevant finding. To ensure a continuously high standard in the practice of prescribing antimicrobials, good documentation that enables all healthcare workers to understand why an antimicrobial is administered is of high importance [17]. Documenting a reason in a patient’s notes indicates that the prescriber put thought and reasoning into the prescription and helps others to better understand and evaluate the prescription, as well as effectively modify it when necessary [18,19].

In recent years, the concept of bundle strategies has been integrated into medical practice including AMS activities [20,21]. For AMU, one of the most effective elements of such a bundle strategy is the use of checklists [22,23]. Our data corroborated this by demonstrating that the existence of a checklist for AMU at the hospital level was associated with > 11 % reduction in the rate of inadequate AMU.

Another interesting finding of our multivariable regression analysis was that the variable tertiary care hospital type significantly reduced the likelihood of adequate antimicrobial applications and significantly increased the likelihood of inadequate applications. This may in part be attributable to differences in patient populations. Patients treated in tertiary care hospitals generally suffer from more severe diseases and, as a consequence, receive more complex treatments [24]. Furthermore, tertiary care hospitals usually employ a higher number of medical doctors whose rotations are more frequent, which could lead to a higher degree of discontinuity in diagnosis and treatment. Another parameter significantly associated with lowering the rate of adequate AMU was high bed occupancy. This finding suggests that a higher workload due to a higher density of patients may lead to less adequate antimicrobial prescribing behaviour, which is possibly less thoughtful and more rushed. A contributing factor may be the link between patient overcrowding (i.e. high bed occupancy) and a more frequent occurrence of HAI [25], which as a result leads to more complicated treatment regimens.

As a secondary objective, this study sought to describe of the state of AMS implementation in German hospitals. Our survey revealed that, as of 2016, less than a third of participating hospitals had designated staff for AMS and only nine hospitals reported dedicating one or more FTE for AMS, which confirms the discrepancy between recommendations [26] and their current implementation. When compared with other European countries [27,28], we consider the current AMS staffing situation and state of implementation of AMS measures in Germany critical and insufficient, at least in the participating hospitals. This is relevant because the training of AMS experts and installation of interdisciplinary AMS teams have shown to improve antimicrobial prescription practices [29].

The German Protection against Infection Act requires hospitals to evaluate and interpret their antimicrobial consumption at least annually and, if necessary, to implement measures to improve consumption accordingly [5]. Over 60% of participating hospitals in our survey reported adherence to this requirement, with also over 60% providing regular feedback on AMU to frontline healthcare workers. This suggests a steady increase in adherence compared with earlier studies [8,30]. Surprisingly, only 38% of hospitals reported participation in structured surveillance networks (regional or national) for antimicrobial consumption, although these are easily accessible and participation is free of costs in Germany [31]. We found this to be an area for potential improvement.

At the facility level, training and education for AMU, as well as local guidelines on empirical antimicrobial treatment, are important factors for improving AMU quality [4,32]. However, data on how healthcare facilities, and in particular acute care hospitals, adhere to these recommendations are scarce. Our data revealed considerable opportunities for improvement, as only 17% of hospitals regularly undertook AMU training and only around a third of participating hospitals had established AMU audits. Our data suggest that the implementation of AMS measures in Germany, as defined by national recommendations [7], is still far from being achieved.

### Limitations

Our study had several limitations. The data used for our analyses were chosen with the objective of identifying structure and process parameters to evaluate antimicrobial prescriptions. The PPS that was conducted in 2016 was not primarily intended for such analyses. Instead, its primary focus was to estimate the prevalence of patients with HAI. However, other variables collected in the PPS allow for careful secondary analyses, as they provide valuable information on structural characteristics of participating hospitals. Another important limitation was that data collection in participating hospitals was performed by a heterogeneous group of professionals with substantial differences regarding experience in conducting surveillance and, more importantly for our analysis, differences in knowledge about antimicrobial prescription. Therefore, inconsistencies in data collection and recording that might confound the data cannot be excluded. However, by ensuring that data collectors were trained systematically before the survey, the data should have gained robustness and consistency with regard to the definitions and methodology applied. In order to categorise the recorded AMU into adequate and inadequate applications, we had to choose parameters and criteria from the limited data available. Furthermore, for the majority of antimicrobial applications recorded, such a categorisation was not possible; these applications were not attributed to either group, but remained undefinable.

We consider the number of included hospitals (n = 218), although not drawn from a representative sample, to be a strength of our study, which allows for careful extrapolations to the national level.

## Conclusions

This study demonstrated the important role of documentation as a factor for improving the quality of AMU. Contrarily, the variables tertiary care hospital type and high bed occupancy were associated with a decrease in adequate AMU, which indicates that a higher workload may be a barrier for the prudent use of antimicrobials. The results also illustrated deficits in the implementation of AMS in German acute care hospitals, in particular with regard to AMS staffing, training for AMU and participation in networks for antimicrobial consumption, which should be tackled. Future studies should focus on novel approaches to utilise point prevalence data to evaluate antimicrobial prescription practices and barriers to successful AMS implementation.

## Supplementary Data

178 Supplement
