# Supplementary material for: The quality of antimicrobial prescribing in acute care hospitals: results derived from a national point prevalence survey, Germany, 2016
Source: Euro Surveill. 2019 Nov 14;24(46):1900281. doi: 10.2807/1560-7917.ES.2019.24.46.1900281 (PMC6864975; doi:10.2807/1560-7917.ES.2019.24.46.1900281)
Supplement: Supplement [file 19-00281_AGHDASSI_Supplement.pdf]

Supplementary material – Univariable analyses

This supplementary material is hosted by *Eurosurveillance* as supporting information alongside the article 'The quality of antimicrobial prescribing in acute care hospitals: results derived from a national point prevalence survey, Germany, 2016' on behalf of the authors who remain responsible for the accuracy and appropriateness of the content. The same standards for ethics, copyright, attributions and permissions as for the article apply. *Eurosurveillance* is not responsible for the maintenance of any links or email addresses provided therein.

Table S1 Results of the univariable linear regression analysis for the outcome rate of adequate antimicrobial applications per 100 definable antimicrobial applications of 218 hospitals that participated in the survey

Outcome	Parameter	p-value	Regression coefficient	95% confidence interval
Rate of adequate antimicrobial applications per 100 definable applications	Hospital size ≥ 300 beds	0.002	-5.69	-9.23; -2.15
	Bed occupancy (%) on the day of the point prevalence survey	0.001	-0.26	-0.41; -0.11
	Bed occupancy (%) as a yearly mean	0.000	-0.35	-0.52; -0.18
	Tertiary care hospital type	0.003	-7.33	-12.08; -2.58
	Primary care hospital type	0.072	3.31	-0.29; 6.91
	Secondary care hospital type	0.454	-1.75	-6.36; 2.85
	Specialised hospital	0.092	5.10	-0.84; 11.04
	Other/Unknown hospital ownership	0.314	-3.12	-9.21; 2.97
	Public hospital ownership	0.193	-2.39	-6; 1.22
	Private (not for profit) hospital ownership	0.577	1.13	-2.85; 5.1
	Private (for profit) hospital ownership	0.044	5.32	0.14; 10.5
	Blood cultures per 100 patient-days (per increase of 1)	0.016	-1.23	-2.23; -0.23
	Stool samples for *Clostridioides difficile* infection (CDI) per 100 patient-days (per increase of 1)	0.053	-3.50	-7.04; 0.04
	Participation in surveillance network for CDI	0.224	-2.24	-5.87; 1.38
	Participation in surveillance network for antimicrobial consumption	0.764	-0.57	-4.29; 3.16
	Participation in surveillance network for antimicrobial resistance	0.691	-0.84	-4.99; 3.31
	Guideline for antimicrobial use	0.926	0.19	-3.85; 4.23
	Audit for antimicrobial use	0.371	-1.74	-5.55; 2.08
	Bundle for antimicrobial use	0.028	4.15	0.45; 7.84
	Checklist for antimicrobial use	0.086	4.92	-0.7; 10.53
	Feedback of data on antimicrobial use	0.949	0.12	-3.65; 3.9
	Surveillance of antimicrobial use	0.965	0.08	-3.75; 3.92
	Training for antimicrobial use	0.338	2.33	-2.46; 7.12
	Prevalence of patients with antimicrobial use (per increase of 1%)	0.499	0.07	-0.13; 0.26
	Documentation of a reason for antimicrobial use in the patient notes (per increase of 1%)	0.015	0.084	0.02; 0.15
	Percentage of hospital beds with post-prescription review of antimicrobials within 72 hours (per increase of 1%)	0.822	0.43	-3.32; 4.17
	Designated staff for antimicrobial stewardship present	0.108	-3.27	-7.26; 0.73

Table S2 Results of the univariable linear regression analysis for the outcome rate of inadequate antimicrobial applications per 100 definable antimicrobial applications of 218 hospitals that participated in the survey

Outcome	Parameter	p-value	Regression coefficient	95% confidence interval
Rate of adequate antimicrobial applications per 100 definable applications	Hospital size ≥ 300 beds	0.512	0.93	-1.87; 3.74
	Bed occupancy (%) on the day of the point prevalence survey	0.355	0.06	-0.06; 0.18
	Bed occupancy (%) as a yearly mean	0.006	0.19	0.05; 0.33
	Tertiary care hospital type	0.028	4.17	0.45; 7.89
	Primary care hospital type	0.330	-1.39	-4.2; 1.42
	Secondary care hospital type	0.818	0.42	-3.16; 3.99
	Specialised hospital	0.166	-3.26	-7.87; 1.36
	Other/Unknown hospital ownership	0.135	3.58	-1.13; 8.29
	Public hospital ownership	0.352	-1.33	-4.13; 1.48
	Private (not for profit) hospital ownership	0.272	1.72	-1.36; 4.8
	Private (for profit) hospital ownership	0.168	-2.83	-6.87; 1.2
	Blood cultures per 100 patient-days (per increase of 1)	0.787	0.11	-0.67; 0.89
	Stool samples for *Clostridioides difficile* infection (CDI) per 100 patient-days (per increase of 1)	0.155	-1.93	-4.6; 0.73
	Participation in surveillance network for CDI	0.973	0.05	-2.77; 2.87
	Participation in surveillance network for antimicrobial consumption	0.190	1.92	-0.96; 4.8
	Participation in surveillance network for antimicrobial resistance	0.811	0.39	-2.83; 3.61
	Guideline for antimicrobial use	0.948	0.10	-3.03; 3.24
	Audit for antimicrobial use	0.059	-2.83	-5.77; 0.11
	Bundle for antimicrobial use	0.026	-3.25	-6.11; -0.38
	Checklist for antimicrobial use	0.031	-4.77	-9.11; -0.43
	Feedback of data on antimicrobial use	0.368	-1.34	-4.26; 1.58
	Surveillance of antimicrobial use	0.540	-0.93	-3.9; 2.05
	Training for antimicrobial use	0.137	-2.80	-6.51; 0.9
	Prevalence of patients with antimicrobial use (per increase of 1%)	0.382	0.07	-0.08; 0.22
	Documentation of a reason for antimicrobial use in the patient notes (per increase of 1%)	0.000	-0.111	-0.16; -0.06
	Percentage of hospital beds with post-prescription review of antimicrobials within 72 hours (per increase of 1%)	0.985	0.03	-2.88; 2.93
	Designated staff for antimicrobial stewardship present	0.419	-1.28	-4.39; 1.83
